# Biochemical Mechanisms Associating Alcohol Use Disorders with Cancers

**DOI:** 10.3390/cancers13143548

**Published:** 2021-07-15

**Authors:** Francisco D. Rodriguez, Rafael Coveñas

**Affiliations:** 1 Department of Biochemistry and Molecular Biology, Faculty of Chemistry, University of Salamanca, 37007 Salamanca, Spain; 2 Group GIR USAL: BMD (Bases Moleculares del Desarrollo), 37007 Salamanca, Spain; covenas@usal.es; 3 Institute of Neurosciences of Castilla y León (INCYL), Laboratory of Neuroanatomy of the Peptidergic Systems, University of Salamanca, 37007 Salamanca, Spain

**Keywords:** alcohol use disorders (AUD), cancer, ethanol oxidative and nonoxidative metabolism, acetaldehyde, reactive oxygen species (ROS), DNA adducts, protein damage, cancer stem cells (CSC), epigenetic changes

## Abstract

**Simple Summary:**

Of all yearly deaths attributable to alcohol consumption globally, approximately 12% are due to cancers, representing approximately 0.4 million deceased individuals. Ethanol metabolism disturbs cell biochemistry by targeting the structure and function of essential biomolecules (proteins, nucleic acids, and lipids) and by provoking alterations in cell programming that lead to cancer development and cancer malignancy. A better understanding of the metabolic and cell signaling realm affected by ethanol is paramount to designing effective treatments and preventive actions tailored to specific neoplasias.

**Abstract:**

The World Health Organization identifies alcohol as a cause of several neoplasias of the oropharynx cavity, esophagus, gastrointestinal tract, larynx, liver, or female breast. We review ethanol’s nonoxidative and oxidative metabolism and one-carbon metabolism that encompasses both redox and transfer reactions that influence crucial cell proliferation machinery. Ethanol favors the uncontrolled production and action of free radicals, which interfere with the maintenance of essential cellular functions. We focus on the generation of protein, DNA, and lipid adducts that interfere with the cellular processes related to growth and differentiation. Ethanol’s effects on stem cells, which are responsible for building and repairing tissues, are reviewed. Cancer stem cells (CSCs) of different origins suffer disturbances related to the expression of cell surface markers, enzymes, and transcription factors after ethanol exposure with the consequent dysregulation of mechanisms related to cancer metastasis or resistance to treatments. Our analysis aims to underline and discuss potential targets that show more sensitivity to ethanol’s action and identify specific metabolic routes and metabolic realms that may be corrected to recover metabolic homeostasis after pharmacological intervention. Specifically, research should pay attention to re-establishing metabolic fluxes by fine-tuning the functioning of specific pathways related to one-carbon metabolism and antioxidant processes.

## 1. Introduction

Alcohol use disorders (AUDs) are chronic diseases where imbibers show compulsive alcohol-seeking and -drinking behavior accompanied by negative emotional states occurring during abstinence periods. The Diagnostic and Statistical Manual of mental disorders, fifth edition (DSM-5), establishes that this diagnosis should meet specific criteria [1]. Excessive alcohol drinking causes diseases and incapacities. The World Health Organization (WHO) reports that harmful alcohol drinking is responsible for approximately 3 million yearly deaths globally. Moreover, ethanol is responsible for approximately 133 million disability-adjusted life years (DALYs), including premature mortality and morbidity [2]. In 2016, of all deaths attributable to alcohol consumption globally, 12.6% were due to cancers, representing approximately 0.4 million deceased individuals.

Furthermore, of the 244 million DALYs attributed to malignant neoplasms, 10.3 million corresponded to cancers attributable to alcohol use [2]. AUDs have a starting pattern of binge consumption. After chronic and intense abuse, the brain’s reward and stress control systems malfunction, and withdrawal, negative emotions, and craving behavior, appear. The brain uses its neurochemical circuitry to adapt and tackle the presence of alcohol [3,4,5,6].

Influential work by Hanahan and Weinberg [7,8] established a conceptual configuration to understand cancer development by defining different hallmarks, for example, the induction of angiogenesis, invasion, and metastasis; resistance to cell death; changes in metabolism; the evasion of immune attack. Alcohol abuse in humans may disturb genetic stability and induce inflammation events that eventually promote cancer in different forms and locations.

This review describes possible mechanisms linking ethanol ingestion with cancer development in different tissues, analyzing ethanol’s effects from its entrance through the digestive tube to its metabolism and its influence on the intermediary and one-carbon unit metabolisms. We evaluate the implication of genetic variants of essential enzymes and other proteins on the damaging action of ethanol. Another section focuses on the generation of protein, lipid, and DNA addition compounds in the presence of acetaldehyde and other oxidative metabolites to ascertain their impact on cancer development. The last section analyzes how ethanol may alter stem cell capabilities in repairing cells and tissues and transition to uncontrolled proliferative pathways. All the molecular changes induced by ethanol on different targets may trigger cancer development, cancer progression, and treatment resistance. Identifying relevant proteinaceous and non-proteinaceous targets will lead to pharmacological interventions to ameliorate or impede the progression of malignant events where harmful alcohol drinking plays a decisive role.

## 2. The Route of Alcohol through the Gastrointestinal Tract in Humans

The dominant alcohol component of alcoholic beverages is usually ethyl alcohol. Additionally, beverages contain other alcohols (such as methanol or 1-propanol) and non-alcoholic congeners (for example, acetone, tannins, and resveratrol) present in different amounts in recorded and unrecorded alcoholic preparations with potential benefit or harm locally and after metabolic transformation. The accompanying substances may exert a coadjuvant or independent role to alcohol [9,10,11]. The heterogeneous compositions of alcoholic drinks add complexity to studying the effects only attributable to ethanol on human health.

Alcohol enters the oral cavity and descends through the esophagus and the gastrointestinal tract, contacting gut epithelia and microbiomes before reaching cells in all organs and tissues. It exerts an irritant effect on the mucosa, and the resident microorganisms absorb and metabolize the substance with consequences both in situ and systemically [12]. In oral human carcinoma samples, Marttila et al. found an increase in the expression of mutated tumor suppressor P53, associated with augmented acetaldehyde production by the microbiome [13]. Alcohol and tobacco alter the composition of the commensal and pathogenic microbiome resident in the oral cavity and gastrointestinal tract, contributing to cancer [14]. Additionally, ethanol and its metabolites and the imbalance in the microbiome populations may affect gut permeability, permitting the passage of bacterial metabolites and endotoxins to the general circulation and inducing inflammation both locally and in distant organs [15]. As a representative example, *Fusobacterium nucleatum*, a bacteria resident in the oral cavity, secretes immune modulators, virulence factors, and microRNAs linked to the initiation and progression of the oral cavity [16,17], esophagus [18], or colorectal [19,20] cancer. In biopsies from patients suffering from colorectal cancer, the expanded bacteria population significantly correlated with alcohol consumption [20]. *F. nucleatum* may also influence tumor growth and metastatic progression in tissues outside the gut, such as the breast. In an in vivo experiment, mice inoculated with lectin Fap-2, expressing *F. nucleatum*, colonize mammary tumors and promote growth and metastasis by blocking the buildup of T cells. The observed effect disappears with antibiotics [21].

The relationship between heavy episodic alcohol drinking (HED) and cancer development depends on covariates (age, diet, smoking, genetic background, environmental conditions, nutrition state, exercise routine, microbiome, and microenvironment), and different mechanisms may confound the impact of a single factor [22,23,24,25,26,27]. The difficulties compound due to the apparent restrictions of experiments on humans. Separating the biochemical routes directly compromised by alcohol and its metabolites is necessary for effective prevention and therapy endeavors. Epidemiological and data-mining studies and animal experiments are also valuable for this purpose [27,28,29,30].

## 3. Ethanol Induces Metabolic Alterations That May Cause or Facilitate Cancer Development

One link that associates chronic alcohol consumption with cancer development is the formation of metabolic products that may induce direct cell dysfunction or cell damage. The metabolic transformation of ethanol stresses many metabolic pathways, which, in turn, may lead to metabolic deficiencies with consequent harmful effects. Next, we review ethanol’s metabolic pathways, emphasizing the role of the metabolites in altering cell function, causing cancer.

### 3.1. Oxidative and Nonoxidative Metabolism of Ethanol

**Figure 1:**
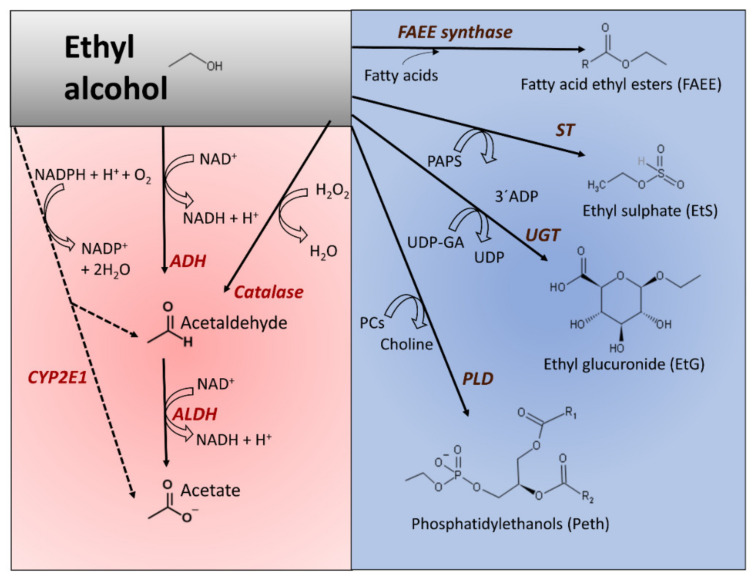
Schematic representation of ethanol oxidative (pink area) and nonoxidative (blue area) metabolism, including metabolites, principal enzymes catalyzing the transformations, and essential coenzymes. Alcohol dehydrogenase (ADH, EC 1.1.1.1); aldehyde dehydrogenase 2 (ALDH, EC 1.2.1.3); cytochrome P450 2E1 (CYP2E11, EC 1.14.14.1); fatty acid ethyl ester synthase (EC 3.1.1.67); PLD phospholipase D (FAEE synthase, EC 3.1.1.4), UDP-glucuronyl transferase (UGT, EC 2.4.1.17), sulfotransferase (ST, EC 2.8.2.2); phosphatidylcholines (PCs); 3’ phosphoadenosine 5’phosphosulphate (PAPS); uridine diphosphate-glucuronic acid (UDP-GA); NAD+, an oxidated form of nicotinamide adenine dinucleotide; NADH, a reduced form of nicotinamide adenine dinucleotide; NADP, an oxidated form of nicotine adenine dinucleotide phosphate; NADPH (a reduced form of nicotine adenine dinucleotide phosphate. Ethanol is oxidized to acetaldehyde by the action of different enzymes. Acetaldehyde is mainly oxidized to acetate by ALDH2. The dashed arrows indicate that CYP2E1 also oxidizes acetaldehyde to acetate. Some of the reactions are reversible in vitro; however, we emphasize the movement of ethanol’s metabolic transformation in vivo [31,32,33,34,35,36,37,38,39,40,41,42,43,44,45,46,47].

The ingestion of ethanol activates metabolic pathways that move electrons across metabolites (oxidative metabolism) or incorporate ethyl alcohol into other chemical structures (nonoxidative metabolism). Figure 1 shows the principal pathways of ethanol metabolism in humans. Due to the low lipid:water partition coefficient of ethanol and its capacity to passively diffuse through biological membranes (determined by the concentration gradient), it reaches an equilibrium concentration influenced by the tissues’ water content, mass, and blood flux supply [31].

The oxidative metabolism of ethanol predominates over nonoxidative conversions. Ethanol oxidates to acetate in a two-step process, sequentially catalyzed by alcohol dehydrogenase (ADH, EC 1.1.1.1) and aldehyde dehydrogenase (ALDH, EC 1.2.1.3). The final product is acetate (Figure 1). The main enzyme responsible for converting ethanol into acetaldehyde is ADH. The reaction uses NAD^+^ as a coenzyme that reduces to NADH, whereas ethanol oxidates to acetaldehyde. ADH is widely present in nature, from bacteria and thermophilic archaea [48] to eucaryote [49]. In mammals, ADH is a family of cytosolic enzymes that shows broad substrate specificity and is present in many tissues [50]. The reaction catalyzed by mammalian ADH requires zinc to stabilize the enzyme’s active center’s essential amino acid residues. Once ingested, gastric ADH oxidates some alcohol and, depending on several factors (sex, enzyme variants, empty stomach, or accompanying medication), the proportion of oxidized alcohol may vary. From the stomach and the small intestine, alcohol is absorbed into the blood and distributed into different tissues. The oxidation of ethanol to produce acetaldehyde mainly occurs in hepatocytes. The reaction consumes the redox coenzyme NAD^+^; therefore, the NADH/NAD^+^ proportion increases.

The fraction of acetaldehyde obtained by other enzymes’ catalytic activity, namely catalase (EC 1.11.1.21) and CYP2E1 (cytochrome P450 2E1, EC 1.14.14.1), represents a small fraction of the total. Catalase is a peroxidase that works in the presence of hydrogen peroxide and ethanol to produce acetaldehyde and water (Figure 1). Although this heme-containing enzyme is present in many tissues, it does not represent the main route for alcohol elimination [51,52]; however, its role in brain tissue is relevant [47]. CYP2E1 belongs to the microsomal ethanol oxidizing system (MEOS) [53]. It also localizes in mitochondria [35]. Where oxygen is present, the enzyme oxidizes ethanol to acetaldehyde and reduces oxygen to water [53]. CYP2E1 has a heme pocket, the polarity of which plays an essential role in its mechanistic adaptation to oxidate alcohol when alcohol concentration increases [54]. The catalytic efficiency of CYP2E1 is very low, showing a higher value for Km compared to ADH. This enzyme also acts on other xenobiotic chemicals, such as anesthetics and halogenated hydrocarbons [55,56]. Additionally, CYP2E1 oxidizes acetaldehyde to acetate with a higher kcat/Km value than that observed in ethanol’s oxidation to acetaldehyde [57]. One consequence of the activation of CYP2E1 by ethanol is the accelerated retinol inactivation, eventually affecting cell proliferation [58].

Two molibdo-flavoproteins, xanthine oxidoreductase (EC 1.17.3.2) and aldehyde oxidase (EC 1.2.3.1) may also oxidize acetaldehyde and propagate reactive oxygen species [59].

Acetaldehyde rapidly converts into acetate, where active ALDH is available. This reaction also reduces NAD^+^ to NADH and increases the index NADH/NAD^+^ (Figure 1). Human ALDHs are a family of enzymes with different preferred substrates and subcellular locations [60]. The isozyme ALDH2 is abundant in liver cells, localizes in mitochondria, and is responsible for acetaldehyde oxidation with a Km value in the lower micromolar range [34]. Its contribution to ethanol removal, ALDH2, also participates in other detoxifying pathways, including eliminating endogenous aldehydes and other metabolites [60,61]. The liver feeds the acetate to other tissues, where it converts to acetyl-CoA, which eventually fully oxidates to carbon dioxide through the citric acid cycle.

**Figure 2:**
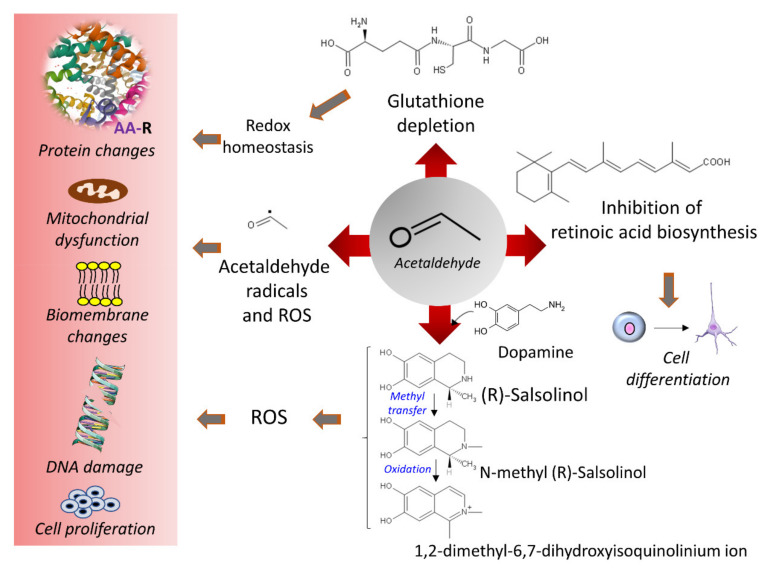
Mechanisms of acetaldehyde cell damage. Due to its reactivity, acetaldehyde covalently alters lipids, DNA, and proteins (for example, histones) by forming adducts. Subsequently, protein damage and DNA damage occur. Acetaldehyde interferes with the metabolism of other compounds such as retinoic acid, hampering cell differentiation. It facilitates the formation of toxic metabolites, including salsolinol and its products, which may damage DNA structure by forming reactive oxygen species (ROS). The figure includes representations of a human nucleosome (Protein Data Bank, code 3AFA). Pyramidal cell drawing: doi.org/10.5281/zenodo.3926221 (accessed on 14 July 2021); DNA representation: doi.org/10.5281/zenodo.4012404 (accessed on 14 July 2021) [59,62,63,64,65,66,67,79,80,81,82,83,84,85].

Acetaldehyde is not only a metabolite of alcohol oxidation but is also present in alcoholic beverages, viands, and tobacco smoke and is a chemical resulting from alcoholic fermentation [62]. Its electrophilic character facilitates reactions with different molecules to produce adducts or secondary products, such as salsolinol, when it condensates with dopamine [63] (Figure 2). Salsolinol may cause DNA damage through reactive oxygen species [64]. The formation of protein adducts by acetaldehyde reacting with amino groups (for example, ε-amino groups of lysine residues) and forming Schiff bases alters many proteins’ structures and functions, including the histones of the nucleosome core [65]. Acetaldehyde also interferes with retinoic acid metabolism and disrupts retinoic acid signaling with consequent alteration in gene expression and cell differentiation processes [66,67]. Acetaldehyde may form DNA adducts that impair proper DNA function if not eliminated or repaired [68,69,70] (Figure 2). In vitro studies with human lung fibroblasts indicated that acetaldehyde induces clastogenic effects with detected DNA breaks at telomeric regions, arresting cells at the G2/M phase and negatively affecting cell viability [71]. The increase in acetate units resulting from ethanol’s active oxidative metabolism, accompanied by food intake restriction, depleted glycogen reserves, and increased NADH concentrations, favors ketone bodies’ synthesis from acetate.

Acetaldehyde depletes glutathione (GSH) and, hence, intracellular redox eucrasia. Different mechanisms may participate: sequestering of GSH pools by non-enzymatic binding [72], reduction in GSH peroxidase (EC 1.11.1.9) activity [73], conjugation with GSH metabolites, such as cysteinyl glycine [74,75], or disruption of the transsulfuration pathway [76]. However, in a murine experimental model of forced GSH depletion obtained by disrupting a glutamate-cysteine ligase modifier (*GCLM*) gene, Chen et al. [77] showed that low levels of GSH protected liver tissue against the insult of ethanol by adapting the metabolic flux of several compounds to resist alcohol insult. Hence, the availability of GSH needs precise modulation to manage volatile metabolic environments.

Among the agents classified by the International Agency for Research of Cancer (IARC), monographs, ethanol, and acetaldehyde appear in group 1 (carcinogenic to humans) [78].

Several enzymes catalyze ethanol’s nonoxidative incorporation into different molecules, including glucuronic acid, sulfate, phosphatidylcholine, or fatty acids (Figure 1, blue-colored panel). These reactions quantitatively represent a minority compared to the oxidative pathways but may have pathological significance due to the modification of metabolism homeostasis [37]. Furthermore, the determination of these alcohol metabolites in different tissues may help ascertain alcohol consumption and unveil unreported alcohol drinking [86].

The transphosphatidylation reaction, prevailing over hydrolytic activity, uses phospholipids, preferentially phosphatidylcholine and ethanol, to produce phosphatidyl ethanol (Peth). The phospholipid forms after alcohol exposure [33,36] by the intervention of phospholipase D (PLD, EC 3.1.4.4) [36,39]. Consequently, Peth, an anionic phospholipid, interferes with phosphatidic acid (PA) synthesis, and its buildup in cell membranes induces changes that may affect membrane-bonded events (cell adhesion, cell signaling, cell trafficking, or cell proliferation) [87,88].

The enzymatic esterification of ethanol and fatty acids generates fatty acid ethyl esters (FAEEs) in different tissues after alcohol exposure [46,89]. Two catalysts participate in this synthesis: FAEE synthase (EC 3.1.1.67) conjugates ethanol with free fatty acids, and (-CoA ethanol-O-acyltransferase (AEAT, EC 2.3.1.84) esterifies acyl chains from acyl-CoA to ethanol [40,89,90]. Carboxyl ester lipase (CEL, EC 3.1.1.3), acting on triglycerides and ethanol, is another source of FAEE, following an ethanolysis (instead of hydrolysis) reaction mechanism [41,42]. In tissues where the oxidative capacity is low, the formation of nonoxidative products, including FAEE, can be relevant, as they may amass in mitochondrial membranes and alter their function [91]. The accumulation of FAEE may also change the activity of key enzyme activities. For example, although it may not appear as the only mechanism, it disrupts the work of adenosine monophosphate-activated protein kinase (AMPKα), a sensor and keeper of energy equilibrium [92]. FAEE leads to the deterioration of cell bioenergetics in human pancreatic acinar cells and causes cell damage since the activation of AMPKα with 5-aminoimidazole-4-carboxamide ribonucleotide (AICAR) attenuated FAEE formation, improved mitochondrial bioenergetics, and alleviated inflammatory responses [93].

Broadly expressed UDP-glucuronosyltransferases (EC 2.4.1.17) catalyze the conjugation of ethanol with glucuronic acid by using 5’-uridine diphosphate (5’-UDP-glucuronic acid) as substrate donor to generate ethyl glucuronide (EtG) as an end product [94]. Sulfotransferases (STs, EC 2.8.2.2) activate the sulfonation of ethanol with 3’-phosphoadenosine-5’-phosphosulfate (PAPS) to produce ethyl sulfate [95]. Some reports established a putative biological role of EtG and other glucuronide conjugates in the activation of Toll-like receptor proteins [38] or the in vitro association of EtG with increased oxidant stress in red blood cells [96]. However, we lack in vivo evidence that both chemicals may have salient biological significance [37]. Because they are water-soluble excretion metabolites, they may serve as biomarkers of alcohol consumption and abstinence vigilance due to their detection after ethanol blood concentrations reach zero value [43,97,98].

### 3.2. Imbalanced Proportion [Free NAD^+^]/[Free NADH]

Tunneling ethanol through oxidation pathways leads to a depletion of oxidated NAD at the expense of increasing its reduced form (NADH). Therefore, the ratio of [free NAD^+]^/[free NADH] decreases.

**Figure 3:**
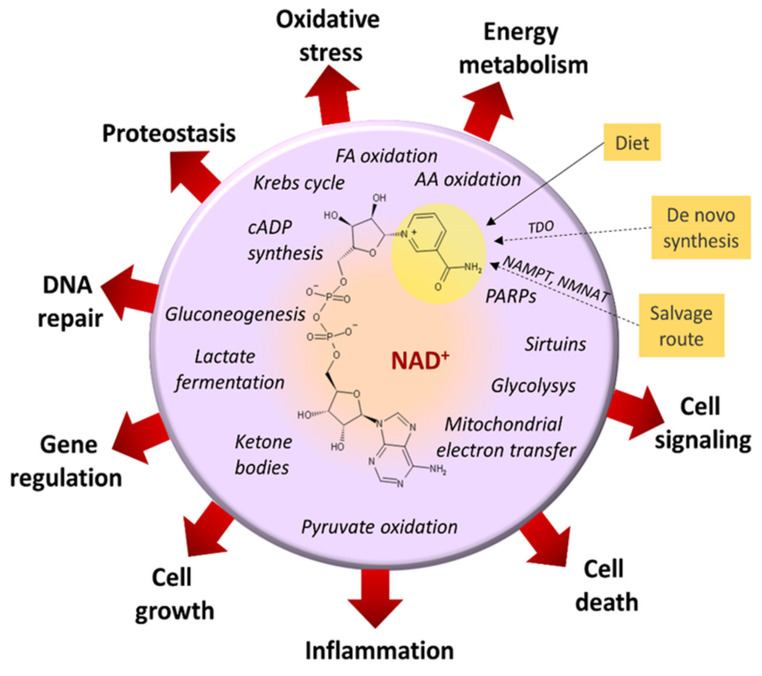
Representation of the metabolic routes affected by an imbalance in the ratio of free coenzymes NAD+/NADH. This imbalance provokes alterations in cell function related to depleted energy state, alteration of DNA repair mechanisms, and the derangement of cell signaling and cell growth with consequent cell death or cell transformation. Dotted arrows indicate several reactions in a metabolic pathway. AA, amino acid; FA, fatty acid; NAMPT, nicotinamide phosphoribosyltransferase, EC 2.4.2.12; NMNAT, nicotinamide mononucleotide adenylyltransferase, EC 2.7,7.1; PARPs, poly(ADP)-ribo. 4.2.30; TDO, tryptophan 2,3-dioxygenase, EC 1.13.11.11 [45,99,100,101,102,110,111].

Hundreds of metabolic reactions (glycolysis, pyruvate fermentation, oxidation of pyruvate, citric acid cycle reactions, mitochondrial electron transport chain reactions, oxidation of amino acids, and fatty acids oxidations) use NAD^+^/NADH as redox coenzymes. Additionally, NAD^+^ is a substrate in other metabolic reactions, such as the deacetylation of histones, polymerization of nucleotides, post-translational modifications of proteins, synthesis of cADP-ribose, or synthesis of NADP+ [45,99,100]. Therefore, NAD^+^-coupled enzymes influence the coenzyme’s bioavailability and energy metabolism [101] (Figure 3). An imbalance in the redox couple concentrations can have consequences that globally affect cell function. An acute outcome is that gluconeogenesis from lactate, serine, and alanine is inhibited [102], and carbohydrate metabolism is significantly compromised. Additionally, alcohol helps switch the metabolism of acetate units to fatty acids storage deposits, aided by the malic enzyme (EC 1.1.1.40) that oxidizes malate to pyruvate and increases reducing equivalents in the form of NADPH [103]. In addition, the altered homeostasis of nicotinamide adenine dinucleotide coenzymes impacts the utilization of nutrients by tumor cells [100].

Depletion of NAD^+^ affects ADP-ribosylation of different protein substrates and histones’ deacetylation by sirtuins (EC 3.5.1.98; EC 2.4.2.31), with consequent cellular stress. Sirtuins are sensors that link energy metabolism with transcriptional regulation and, depending on the context, may behave as mitochondrial tumor suppressors or tumor-promoting agents, as is the case for sirtuin Sirt 4 [104]. Deficits in sirtuin Sirt1 deacylase activity disrupt lipid metabolism and alter specific transcriptional factors, favoring alcohol liver damage progression [105].

The poly(ADP-ribose) polymerase (PARP, EC 2.4.2.30) enzymes also depend on NAD^+^ availability. In experimental models of alcohol-induced steatohepatitis, Mukhopadhyay et al. [106] reported that PARP inhibition restored Sirt1 activity and ameliorated the induced inflammatory and oxidative stress responses. The results associate PARP and Sirt1 actions through the use of NAD^+^ in their reactions.

The free AMP concentration also regulates the enzyme AMPK. This enzyme promotes ATP synthesis by increasing ATP catabolic production and inhibiting ATP anabolic needs. It also regulates several metabolic pathways that maintain a state of metabolic homeostasis and adequate cell function. One consequence of ethanol oxidation is the augmented [free NADH]/[free NAD^+^] proportion and the cell’s energy state. Consequently, free AMP concentrations decay and AMPK activation is blocked. Therefore, the metabolic activities, depending on AMPK’s proper functioning, are affected [44]. Given the essential roles of AMPK in anabolic metabolism, cell growth, proliferation, autophagy, and DNA repair, it plays a central role in carcinogenesis control [107].

NAD^+^ is a chemical beacon regulating many of the enzyme activities responsible for essential metabolic control and disease resilience [108]. Alcohol ingestion may affect cell functioning, as well as other mechanisms, by disabling the delicate balance of [free NAD^+^]/[free NADH] and the energy state of the cell [109] (Figure 3).

### 3.3. Ethanol and Metabolism of C1-Units

**Figure 4:**
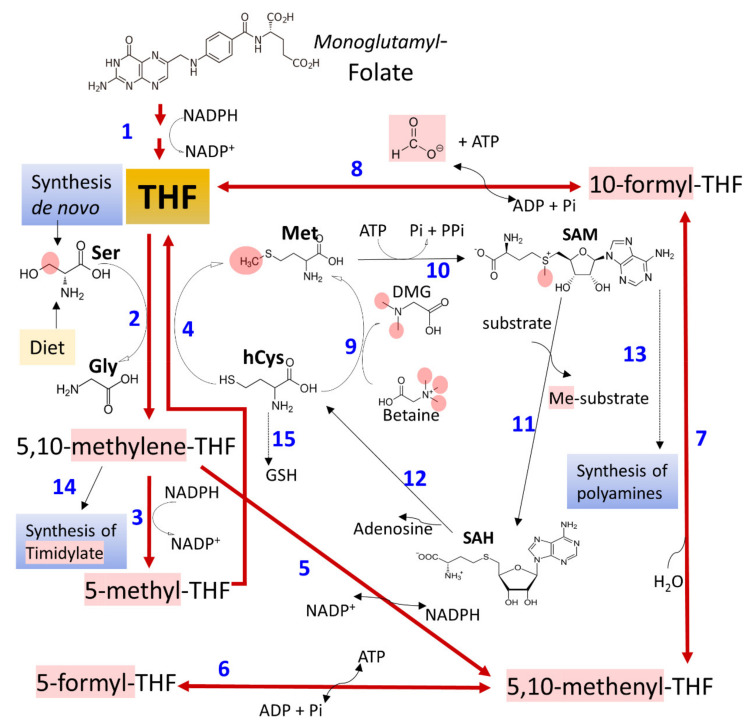
The hub of one-carbon metabolism pathways. The figure depicts the main routes involved in transferring one-carbon modules (highlighted in red) from one metabolite to another. 1, Dihydrofolate reductase, EC 1.5.1.3; 2, Ser-hydroxymethyl transferase, EC 2.1.2.1; 3, methylene THF reductase, EC 1.5.1.20; 4, MS: Met synthase, EC 2.1.1.13; 5, methylenetetrahydrofolate reductase, EC 1.5.1.20; 6, 5-formyltetrahydrofolate cyclo-ligase, EC 6.3.3.2; 7, methyltetrahydrofolate ciclohydrolase, EC 3.5.4.9; 8, formate-tetrahydrofolate ligase, EC 6.3.4.3) 9, betaine: homocysteine methyltransferase, EC 2.1.1.5; 10, Met adenosyltransferase, EC 2.5.1.6; 11, methyltransferases, EC 2.1.1.; 12, S-adenosylhomocysteine hydrolase, EC 3.3.1.1; 13, SAM decarboxylase and other enzymes; 14, thymidylate synthase, EC 2.1.1.45; 15 indicates the transsulfuration pathway where several enzymes participate [76,117,124,128,130,136,137,138,139].

The term C1 metabolism refers to the circuitry of metabolic pathways (Figure 4), essential for cell proliferation and endurance (nucleotide synthesis, Ser Gly and Met metabolism, methylation reactions, synthesis of polyamines, redox reactions, and transsulfuration pathway), which manage the provision of one-carbon modules (methyl groups) and connects with the control of the cellular redox state [76,112,113,114,115,116]. Folate (pteroil-L-glutamate) metabolism distributes one-carbon components through redox reactions. It also couples with metabolic routes where a carbon atom moves between different substrates through transfer reactions essential for cellular proliferation and epigenetic regulation [112,114,115,116]. Many reports analyze valuable information concerning the role of one-carbon metabolism in cell proliferation, differentiation, and growth [112,113,116,117,118,119,120,121].

Several studies report that the chronic ingestion of alcohol affects folate availability and metabolism, not restored after folate’s additional dietary supply [122]. In humans, many mechanisms possibly contribute, including reduced intestinal absorption [122,123,124], augmented renal excretion [125], and the oxidative disruption of the molecule through the effect of acetaldehyde or free radicals derived from their oxidation [126]. Sustained alcohol consumption impairs an intestinal γ-glutamate carboxypeptidase (GCP, EC 3.4.17.11), which hydrolyzes polyglutamate forms of folate to absorbable forms. It also reduces the action of folate binding proteins, which is also essential for absorption [124,127]. Additionally, ethanol may interfere with metabolic pathways, involving the movement of one-carbon units (Figure 4) by affecting essential enzyme activities, such as methionine synthase (MS, EC 2.1.1.13). GSH also influences it since homocysteine is a source for the synthesis of GSH by the transsulfuration route [76,128]. Ethanol-treated micropigs during 14 weeks showed reduced liver methionine synthase activity, lowered S-adenosyl methionine (SAM) and GSH, and elevated plasma concentrations of malondialdehyde (MDA). These findings correlated with detecting acetaldehyde adducts, DNA damage, and liver injury [124]. Methionine deficiency affects the levels of other metabolite pools, including the methyl donor SAM, thus negatively impacting essential methylation reactions [129]. Additionally, 5-methyl-THF accumulates in a trapped pool that cannot convert into other folate forms, as methyleneTHF reductase (EC 1.5.1.20) catalyzes an irreversible reaction (Figure 4). This effect has consequences on different reactions responsible for DNA synthesis and DNA-defect repair [122,130,131]. However, the evidence obtained points to variable impact, depending on the organ exposed. For example, experimental chronic alcohol treatment in rats differentially affects one-carbon metabolism in liver and brain tissue [132].

The relationship of ethyl alcohol with carcinogenesis and tumor progression is complex and manifold. The influence of alcohol on folate metabolism plays a prominent and not yet well-understood role. The altered metabolism may induce malignant transformation, but cell proliferation in tumors requires complete folate metabolism to support the synthetic demands of the transformed cells [26,27,81,133,134,135,136]. Consequently, chemicals designed to target folate metabolism’s crucial steps are essential tools when treating or preventing cancer, whether ethanol or other known etiological factors play their part.

### 3.4. Ethanol and Oxidative Stress

**Figure 5:**
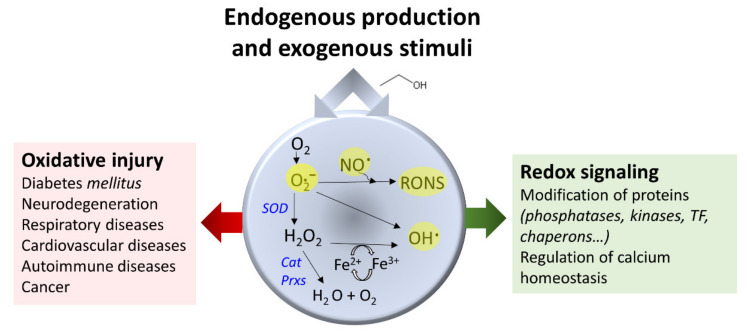
Exogenous agents (for example, ethyl alcohol) and endogenous mechanisms lead to reactive species formation. These species contribute both to physiological signaling and cell damage if the balance breaks. Essential enzymes, including Cat (catalase, EC 1.11.1.21), Prxs (peroxiredoxins, EC 1.11.1.15), and SOD (EC 1.15.1.1), support antioxidant defense activity. TFs, transcription factors; RONS, reactive oxygen and nitrogen species [70,140,141,142,147,157].

Oxidative stress refers to a state characterized by increased production of highly reactive species. Two reactive species classes [140] are, on the one hand, radicals or short half-life molecules with unpaired electrons in their atoms’ outer orbitals (examples include superoxide, oxygen radical, hydroxyl, alkoxy radical, peroxyl radical, and nitric oxide). On the other hand, non-radical molecules (for example, hydrogen peroxide, singlet oxygen, and nitrosyl cation) can easily give rise to radicals (Figure 5). The balance of the concentration of these molecules is paramount to serving cell function since reactive species’ production does not always associate with cell damage, and essential activities depend on the action of these molecules [141,142,143]. These compounds can be intracellularly generated or may have an exogenous origin [140]. Mitochondrial enzymatic activities carried out by the mitochondrial electron transfer chain, monoamine oxidases, and several dehydrogenases are a primary source of reactive oxygen species (ROS) [144,145,146]. Reactive oxygen and nitrogen species (RONS) derive from nitric oxide synthase and NADPH oxidase enzymatic reactions [147,148].

The escalation of acetaldehyde concentration brings together oxidative activities that can produce ROS. Both acetaldehyde and ROS contribute to carcinogenesis by covalently modifying DNA, proteins, and lipids, leading to function losses [149,150]. A few examples below illustrate this assertion. In alcoholics, the catalytic hemoprotein CYP2E1 is a ROS source [35,55,151]. Epithelial gastric cells increased ROS formation after direct exposure to 0.1% (*v/v*) acetaldehyde [82]. Human neurons treated with ethanol showed an elevated synthesis of RONS linked to the activation of NADPH oxidase (NOX, EC 1.6.3.1), xanthine oxidase (XOX, EC 1.17.3.2), and inducible nitric oxide synthase (iNOS, EC 1.14.13.39) via acetaldehyde [152]. In neuroblastoma SH-SY5Y cells, acetaldehyde provoked mitochondrial fragmentation and cell damage in a ROS-formation- and calcium-dependent manner by phosphorylating dynamin-related protein1 (Drp1), a key regulator of mitochondrial fission [84]. In neurovascular tissues, acetaldehyde induces oxidant production enzymes, NADPH oxidase, and iNOS [153]. Synaptosomal membranes isolated from chronically treated rats showed reduced activity of superoxide dismutase (SOD, EC 1.15.1.1), glutathione peroxidase (GPx, EC 1.11.1.9), and catalase, accompanied by changes in lipid composition that altered membrane-bound enzyme function; vitamin E annihilated the observed changes [154]. Alcohol produced increased ROS and triggered apoptosis in gastric epithelial cells by downregulating mitogen-activated protein kinase cascades. Theaflavins, polyphenols with antioxidant properties, alleviated the fluctuations [155]. With ROS formation after ethanol exposure, iron accumulates and may contribute to the oxidative milieu for its ready participation in one-electron transfer reactions [156].

Antioxidant enzymes depict a paradoxical role and may benefit or derange redox homeostasis [157]. They encompass specialized enzymes, reductive compounds, or chelates. For example, the enzyme SOD converts superoxide anion (O2.-) into hydrogen peroxide and water. Catalase reduces two hydrogen peroxide molecules to water and molecular oxygen (Figure 5). Glutathione peroxidases reduce many substrates, including hydrogen peroxide, by the concomitant oxidation of reduced glutathione to oxidized glutathione (GSSG). Peroxiredoxins (EC 1.11.1.15) oxidize their N-terminal Cys residue to reduce hydrogen peroxide [157,158].

The dysregulation of antioxidant barriers may also account for the harmful effect of acetaldehyde. Researchers found a diminished serum antioxidant activity in rats treated with alcohol (an oral diet containing 15% *v/v*) for two months [159]. The direct exposure of endothelial cells of human origin to alcohol and acetaldehyde increases RONS production and augments SOD and catalase activities [160]. Many factors play a part in gastric and colon cancer; the generation of ROS caused by the suppression of enzymes with antioxidant capacity contributes significantly [161]. Chronic, but not acute, ethanol exposure induces an oxidative milieu that may facilitate the progression from chronic hepatitis to cirrhosis and hepatocellular carcinoma [162]. Many studies report contradictory results regarding how and to what extent alcohol influences the cells’ antioxidant barrier status [163,164,165,166,167]. Different results may reflect the different experimental approaches (mostly related to the amount and pattern of exposure and the experimental specimen under analysis).

Another aspect worth considering is that ethanol can promote cancer development in different tissues and contribute to cancer cell survival. In malignant colon cells, ethanol promotes cell survival. It favors a more malignant phenotype in an oxidative environment by activating nuclear factor erythroid 2-related factor 2/heme oxygenase 1 (Nrf2/HO-1) recruitment to the nucleus that eventually provokes the activation of antioxidant enzymes and bolsters malignant cell survival [168].

Additionally, the failure of complete oxidation by ethanol metabolizing enzymes may generate acetaldehyde radicals (ethoxy, hydroxymethyl, or acetyl) with potential toxic effects [59,169].

### 3.5. Gene Variants

Another origin of metabolic changes is genetic variants of enzymes that participate directly in ethanol elimination, exhibit an antioxidant task, or are key in different metabolic routes that maintain cell function. Gene variants relate to sensitivity to alcohol and its metabolites [170,171,172,173].

When analyzing ethanol’s influence as a risk or causal factor of certain types of cancer, one should consider that some gene polymorphisms linked to alcohol metabolism may contribute to maintaining harmful drinking behavior, which sustains and prolongs damage. The expression of some genes and variants may also be affected by alcohol or its metabolites. Other genes or variants not influenced by alcohol can contribute as primary risk elements independently or in an additive or synergistic manner combined with alcohol drinking. Therefore, the dissection of specific mechanisms accountable for direct or selected damage is challenging; however, establishing relationship patterns may clear these interactions and their relevance in delineating prevention and treatment strategies.

Gene variations of two ethanol metabolizing enzymes, ADH and ALDH, may facilitate acetaldehyde accumulation. Some polymorphisms of these two enzymes are associated with the enhanced or attenuated drinking behavior of imbibers. The distribution of these polymorphisms varies among ethnic and geographical groups, adding complexity to the unveiling of their impact on pathologies, including cancer [174,175,176,177,178,179,180]. Cancer cells of the alcohol-metabolizing organs of the gastrointestinal system show activated ADH and attenuated ALDH activities compared to healthy tissues. This observation relates to different isoenzymes acting in cancer cells [177]. Furthermore, evaluating enzyme polymorphisms as a risk for the development and progression of malignancies needs attention within the context of other factors (nutrition, smoking habit, sex, age, diabetes, and obesity) that may also contribute.

Human alcohol dehydrogenases (ADHs) can be arranged into five classes. Class I includes ADH1A, ADH1B, and ADH1C isoenzymes [181], which may form homodimers or heterodimers responsible for almost all ethanol oxidation in the liver [50,182]. Single nucleotide polymorphisms (SNPs) in coding and noncoding regions influence the function or the expression of the corresponding protein, affecting its overall performance [50,183]. ADBH1B variations ADH1B*2 (rs1229984, Arg48His), ADH1B*3 (rs2066702, Arg370Cys), and ADH1C (rs698, Ile350Val, and rs1693482, Arg272Gln) manifest higher activity compared to normally functioning alleles, thus accelerating ethanol’s oxidation to acetaldehyde [174,184]. However, this effect may disappear if a robust form of ALDH acts on increasing acetaldehyde [50].

Human ALDHs form a family of nineteen members. Only three of them, ALDH1A1, ALDH1B1, and ALDH2, are relevant for acetaldehyde oxidation to acetate [50,185]. Drinking patterns and alcohol metabolism are affected by the allele variation rs671 (Glu487Lys) of isoenzyme ALDH2, ALDH2*2, observed mainly in Asian populations. The amino acid change (acidic to basic) blocks the enzyme, and, consequently, acetaldehyde accumulates. The variation may protect the carrier from excessive alcohol ingestion to avoid the unpleasant effects caused by acetaldehyde [186,187].

The combination of more effective ADH variants and deficient ALDH activities favors the exposure of cells to acetaldehyde. The epidemiological analysis of specific human communities relates to these gene variants with a higher risk of gastrointestinal tract cancer [188,189,190,191,192]. Additionally, polymorphisms of ethanol-metabolizing enzymes and gastric colonization by bacteria (*Helicobacter pylori* and others) contribute to augmented acetaldehyde concentrations in the stomach and colon [161].

Cytochrome CYP2E11 belongs to the Cytochrome P450 subfamily of enzymes. The single nucleotide variation (SNV) of the gene *CYP2E11*, rs2031920, associates with colorectal cancer risk [193]. Polymorphisms rs2031920 and rs6413432 associate with prostaglandin G/H synthase 2 gene (*PTGS2*) polymorphisms for lung cancer risk in specific human groups [194] (Table 1). Two variants, rs72559710 (Arg76His) and rs55897648 (Val389Ile) [195] may affect the function of the enzyme, although its relationship with cancer risk is not well-defined and needs more in-depth analysis [55].

Additional enzyme variants may associate with cancer risk. Compared to the most common genotype, the homozygous rs1801133 (Ala222Val) variant of the enzyme methylenetetrahydrofolate reductase (MTHFR) shows reduced activity. Many studies analyzed the link between excessive alcohol drinking and this variant of *MTHFR* with risk of head and neck, gastrointestinal tract, or lung cancer in different human populations [174,198,214,216,217,218,219,220,221,222]. SOD variants rs4998557 and rs4880 (Ala16Val), *Helicobacter pylori* infection history, and alcohol drinking correlate with gastric cancer risk [209]. The *SOD* rs4880 variant is associated with the epidermal growth factor receptor (EGFR) Leu858Arg mutation in patients with non-small-cell lung cancer and primary brain tumors [208] (Table 1).

Lipid metabolism is also affected by alcohol. In liver tissue, fat accumulates, leading to liver steatosis and fibrosis [223]. An enzyme associated with these processes is patatin-like phospholipase domain-containing 3 or adiponutrin (PNPLA3, EC 2.3.1.51), a protein that catalyzes coenzyme A (CoA)-dependent acylation of 1-acyl-sn-glycerol 3-phosphate to produce phosphatidic acid (PA). Additionally, it shows triacylglycerol lipase (EC 3.1.1.3) and CoA-independent acylglycerol transacylase (2.3.1.147) activities [224,225]. Genetic variation in *PNPLA* gene rs738409 is associated with steatosis and fibrosis in individuals with alcoholic liver disease [69,211,212]. The occurrence of single nucleotide variants rs738409 in the *PNPLA* gene and rs58542926 in transmembrane 6 superfamily member 2 gene (*TM6SF2*) associate with the risk of hepatocellular carcinoma, whereas variant rs4607179 of hydroxysteroid 17-beta dehydrogenase gene (*HSD17B13*) reduces the risk [213]. In a genome-wide association study, variants rs738409 of *PNPLA* gene and rs4607179 of *HSD17B13* gene showed significant association with the appearance of alcohol-associated liver cirrhosis, and rs374702773 of Fas-associated factor family member 2 gene (*FASF2*) showed a protective association [206].

Additional gene variants of proteins (phosphatases, phospholipases, kinases, receptors, and DNA-repair enzymes) are statistically associated with developing different types of cancer in the context of alcohol drinking [178,210,226,227,228].

Polymorphism studies are relevant to ascertaining cancer risk in certain tissues and specific populations. The analysis of polymorphisms in selected alcoholic cohorts also provides essential information. Nevertheless, we need broader analysis and studies that link these relationships with experimental evidence at the molecular level. The task is not easy but is necessary. Alcohol may represent an independent risk factor that may act in an additive or synergistic manner with the polymorphic background of certain enzymes found in selected human communities. In this regard, genome-wide association studies (GWAS) and gene-environment-wide interaction studies (GWIS) [178] may serve, together with biochemical analyses, to offer a clearer picture.

### 3.6. Ethanol and Cancer Development

Humans usually ingest alcohol per os, exerting a direct irritant impact on mucoses. Additionally, resident oral and gut microbiota populations destabilize and locally metabolize the substance to generate acetaldehyde. Local effects of ethanol include changes in the permeability of the intestinal wall and the pass of bacterial endotoxins and metabolites that may induce inflammation. Once ethanol reaches the general circulation, it arrives at cells in different organs, but the liver is the central station of arrival. Cells may metabolize ethanol through oxidative and nonoxidative pathways, producing noncommon molecules that alter the intermediary metabolism, break the proportion of essential coenzymes NAD^+^ and NADH, detour energy production mechanisms, alter substrate fluxes in one-carbon unit pathways, and generate free radicals. These transformations may enhance oncogenic transformation by damaging DNA. To the modifications mentioned above, gene variants affecting a diversity of enzymes and proteins add complexity to the effects of ethanol on human health. The elements contributing to the described framework of alcohol capacities may facilitate cell, tissue, and organ suffering, leading to disease. Poblational studies identify alcohol and its metabolites as necessary contributors to cancer development. This association receives support from basic research using in vivo and in vitro models analyzing the impact of ethanol on specific targets. Unfortunately, we cannot precisely define ethanol as a specific agent leading to carcinogenesis, but indirect evidence is overwhelming as we relate in this review. According to the defining hallmarks of cancer [8], alcohol metabolism may result directly in carcinogenesis or accompanied by other agents and mechanisms due to its capacity to induce inflammation, DNA damage with subsequent oncogenic activation, and protein/enzyme malfunction.

## 4. Damage of DNA and Proteins and Epigenetic Shifts

Direct or indirect alcohol metabolism products may react with proteins, lipids, and DNA, affecting their architecture and function with consequences on the activation of cell proliferation or inhibition of tumor-suppressor mechanisms [26,27,62,68,79,83,150,229,230,231]. One of these notable metabolites is acetaldehyde. Additionally, reactive oxygen and nitrogen species, lipid peroxidation products [68], and folate and estrogens’ altered metabolism play a significant role [26].

**Figure 6:**
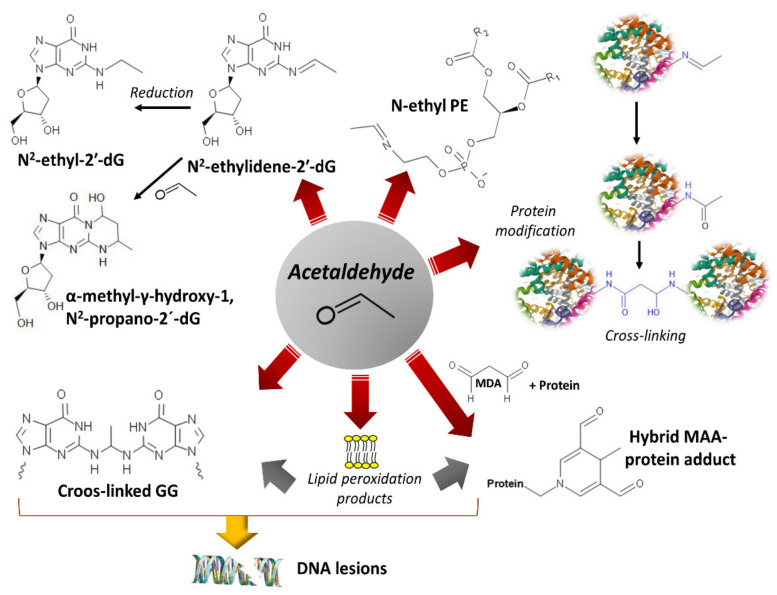
Protein, DNA, and phospholipid adducts are generated by the reaction of electrophilic acetaldehyde with nucleophilic groups. Additional secondary transformations may occur. dG, deoxyguanosine; GG, two contiguous deoxyguanosine on a DNA strand; MDA, malondialdehyde; MAA, malondialdehyde acetaldehyde; PE, phosphatidylethanolamine. The figure includes colored representations of a human nucleosome (Protein Data Bank, code 3AFA). DNA figure: doi.org/10.5281/zenodo.4012404 (accessed on 14 July 2021) [26,62,80,82,83,85,149,229,231].

Acetaldehyde is an electrophile that reacts with nucleophiles to generate covalent adducts with lipids, proteins, and nucleic acids (Figure 6).

Although acetaldehyde rapidly oxidizes to acetate by ALDH, its concentration can reach significant levels in individuals carrying poorly active ALDH2 variants, facilitate the formation of adducts [65,232], and augment cancer risk [200]. Experimentally increased acetaldehyde concentrations obtained in ALDH knockout mice exposed to ethanol elicited an increase in acetaldehyde-DNA adducts [233]. Furthermore, the disruption of DNA repair mechanisms, such as the tumor suppressor OVCA2 [234] or tumor suppressor p53 [235], dramatically influences acetaldehyde tolerance. The aldehyde reacts with amino groups in proteins (ε-amino of lysines and α-amino of the amino acids at the N-terminus). Firstly, unstable, reversible Schiff base forms—later, stable adducts—may derive in intrachain and interchain crosslinked structures [83].

Acetaldehyde also forms DNA adducts by reacting with nucleophilic amino groups. Reaction with guanine’s purine ring produces N2-ethylidenedeoxyguanosine, which converts to the reduced, more stable adduct, N2-ethyldeoxyguanosine [65,70,151,236] (Figure 6). Additionally, acetaldehyde can generate intrastrand crosslinked structures between two adjacent guanines and significantly disturb the double helix’s topography [237]. Another DNA adduct, α-methyl-γ-hydroxy-1, N2-propane-2’-deoxyguanosine (detected as an open aldehyde or closed-ring form), is the product of the reaction of acetaldehyde with the guanine ring occurring in a basic environment (e.g., arginine and lysine basic groups on the neighbor histones) [68,238] (Figure 6). This guanosine adduct also appears after the nucleophilic attack of prop-2-enal (a lipid peroxidation product) [239].

**Figure 7:**
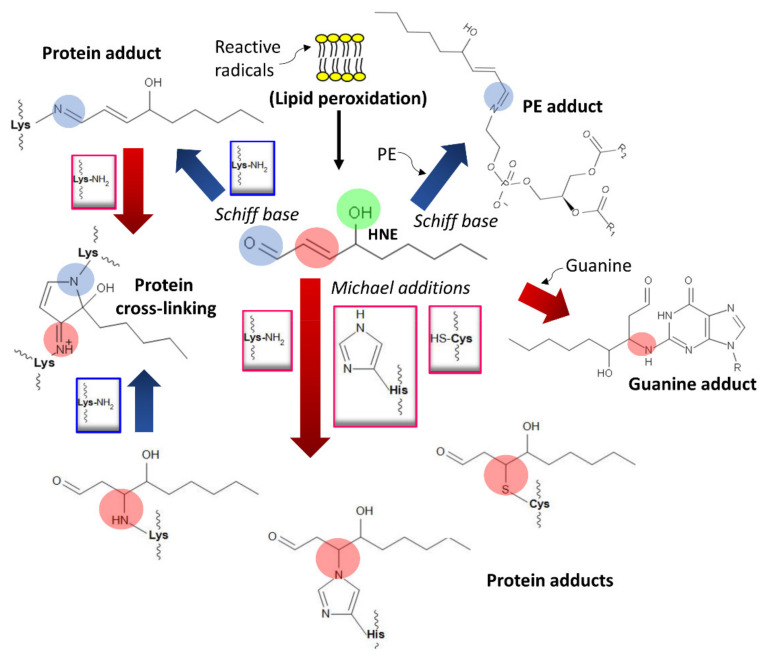
Protein adducts are generated by the reaction of amino, imidazole, and thiol groups of amino acids with reactive carbons of 4-hydroxy-2-trans-nonenal (HNE) through Schiff base formation or Michael additions to the C2=C3 double bond. HNE may react with lipids and DNA to generate adducts. Additional secondary reactions may occur between the carbonyl and the hydroxyl groups. Colored circles represent reactive groups within HNE that undergo different redox reactions. PE, phosphatidylethanolamine [79,151,230,241,242,243].

High concentrations and chronic consumption of ethanol activate CYP2E11 with consequent local accumulations of ROS [55], causing the lipid peroxidation of biomembranes that yield reactive aldehydes—4-hydroxynonenal, prop-2-enal, 4-oxo-2-nonenal, and malondialdehyde—and their epoxides from oxidized lipids containing polyunsaturated fatty acids (PUFAs) [68,240,241]. These compounds non-enzymatically react with proteins through Michael additions and Schiff base formation and generate lipid and DNA adducts (Figure 7) [241,242].

They also form adducts with membrane lipids, such as phosphatidylethanolamine or phosphatidylserine, to produce phospholipids exhibiting modified solubility that influences the membrane’s biophysical properties [81,244] (Figure 7). Additionally, acetaldehyde and malondialdehyde (a lipid peroxidation product) may generate malondialdehyde-acetaldehyde (MAA) protein hybrid adducts [83,245,246].

Many adducted proteins are covalently modified after reacting with aldehydes, and they have numerous activities [65]. Covalent modifications may alter function (often by inactivation), depending on the protein’s targeted amino acid(s). Moreover, these modifications block enzymes involved in ethanol metabolism. In this situation, they serve as a control mechanism to regulate ROS and acetaldehyde accumulation and modify drinking behavior [65,80,247]. Protein adducts may trigger an immune response directed against specifically modified epitopes (adduct epitopes) acting as neoantigens [245,248,249,250]. This phenomenon affects the immune responses that are essential for controlling cell damage and extracellular milieu homeostasis.

DNA modifications lead to secondary reactions carried out by exposed carbonyl or hydroxyl groups, which result in intrastrand and interstrand crosslinks and DNA-protein structures crosslinked that obstruct proper function. DNA lesions, if not repaired, propagate as mutations after DNA replication. Specific mutations in genes that code for tumor-suppressor proteins, transcription factors, or proteins responsible for cell cycle surveillance may lead to cell decline or uncontrolled cell proliferation, cell transformation, and malignancy [229,230,235]. Furthermore, the cell’s difficulties in repairing damage by canonical mechanisms amplify damage [68,243]. Analytical assessment of these adducts represents a valuable tool, combined with other biomarkers’ analyses, to better monitor AUD patients and predict reliable disease risk profiles [65].

As a consequence of DNA damage, mutations of oncogenic routes located at the so-called fragile sites accumulate and enhance their activity. Their mutated products compromise DNA repair and trigger the onset of cell transformation by different pathways. Specific oncogenic routes associate with several cancer types and define mutational signatures [251]. Specifically, among these signatures, tumor suppressor p53 (TP53), cullin 3 (CUL3), and nuclear receptor binding SET domain protein 1 (NSD) contribute to profiles related to alcohol consumption of cancer patients [251]. Examples of population studies observe the activation of oncogenic routes such as Kirsten rat sarcoma viral oncogene homolog gene (*KRAS*) [252], tumor suppressor genes adenomatous polyposis coli (*APC*) and *TP53* [13,253], cyclin D1 (*CCND-1*), or matrix metalloprotease 2 (*MMP2*) [254], in different cancers types influenced, at least in part, by alcohol consumption [255]. In some types of cancer, the association is unclear [253].

Epigenetic mechanisms (dependent on noncoding RNA signaling and chromatin modifications) play a significant role in cancer development by silencing tumor suppressor genes or facilitating unstable genomes [256]. Observed modifications (described previously) induced by ethanol in one-carbon metabolism, antioxidant barriers, NADH concentration, noncoding RNAs regulatory function, and altered autophagy mechanisms may affect DNA regulation histone architecture and compromise gene expression [138,257,258,259,260,261,262,263,264]. Other intrinsic and extrinsic factors may determine these epigenetic shifts in a scenario where alcohol may synergize the injury process [265,266,267]. 

This review considers the influence of ethanol on different signaling pathways leading to transformation in cancer stem cells (CSCs) (Section 5).

Table 2 summarizes some targets that may show different sensitivities to ethanol and its metabolites in the context of cancer development. Research efforts should address ethanol’s direct impact on specific biomolecules and the altered metabolic environments arising after ethanol exposure.

## 5. Alcohol, Cancer Stem Cells Theories, and Therapeutic Strategies

Stem/progenitor cells play a pivotal role in the development of organs and the response to tissue injuries. The study of the harmful actions mediated by ethanol and its metabolites (e.g., acetaldehyde and acetate) opens a new and essential research line on the changes operated on stem/progenitor cells [269]. A small number of tumorigenic stem cells (tumor-initiating cells) drive the growth of tumors. Hence, in-depth knowledge of alcohol’s actions on cancer stem cells’ biology may lead to new therapeutic strategies to counteract the occurrence of tumors induced by alcohol [270].

Alcohol generates the ROS responsible for damages in the DNA, biomembranes, and proteins. Additionally, it decreases NAD^+^ concentrations (promoting a decrease in the differentiation and self-renewal of stem cells) and deranges signal transduction pathways [269,271]. ROS control the renewal and differentiation of stem cells [272,273]. Decreases in the intracellular levels of NAD^+^ exert a negative effect on stem cells [269]. Fully functional ALDH activity counteracts the toxic effect induced by acetaldehyde and is involved in stem cell protection, maintenance, and differentiation [269,274]. A high level of aldehyde dehydrogenase in tumor-initiating cells relates to cancer stem cells’ expansion, although this enzyme is not the only contributing factor [269,275].

Epigenetic processes control stem cells (self-renewal and differentiation). Alcohol can alter these epigenetic processes by perturbing the expression of those genes involved in the maintenance, proliferation, and differentiation of stem and progenitor cells [276,277,278]. Alcohol decreased the expression of stem cell markers in stem/progenitor cells (e.g., neural, adventitial, and hematopoietic progenitor cells: hepatic, intestinal, and dental pulp stem cells). It also intercepted cell differentiation, induced DNA damage, and apoptosis, and promoted phenotypic changes. Alcohol also altered the stem cell niche’s microenvironment and disturbed cell fate determination by dysregulating the extracellular matrix deposition and changing the plasma membrane’s fluidity (reviewed in [269]). Fanconi anemia and transforming growth factor β pathways are involved in the maintenance and differentiation of stem cells and alcohol metabolization [15]. The tumor suppressor β2-spectrin increased the tumor suppression mediated by transforming growth factor β [279]. Both β2-spectrin and transforming growth factor β pathways, by controlling the Fanconi anemia DNA repair pathway, are involved in the genomic stability maintenance from genotoxic metabolites. β2-spectrin maintains this stability after alcohol-induced DNA damage, and inflammation and cancer appeared when a defective transforming growth factor β signaling occurred [279].

Ethanol exerts teratogenic effects and is responsible for both fetal alcohol syndrome (FAS) and fetal alcohol spectrum disorders (FASDs) [280]. Ethanol promoted a loss of pluripotency in embryonic stem cells; in these cells, a decreased staining for TRA-1-81 and alkaline phosphatase was observed, but no change in other markers, such as TRA-1-60, SSEA4, and Oct4, was found [281]. Alcohol promoted DNA methylation changes in several chromosome regions and altered genes related to oxidative stress/metabolic processes in human embryonic stem cells [282,283] and induced the differentiation of embryonic stem cells by activating the nuclear transcriptional program controlled by the retinoic acid/retinoic acid receptor gamma complex [280]. During embryogenesis, alcohol exerted a teratogenic effect on neurodevelopment; this may explain, at least in part, the pathology of FASDs [284]. Ethanol reduced the level of purine receptor three associated with pain (P2RX3) in undifferentiated human embryonic stem cells. Still, it increased both P2RX3 mRNA/protein levels in human embryonic stem-cell-derived neural precursor cells. Moreover, it altered the Toll-like receptor and signal transducer and activator of transcription 3 (JAK-STAT) signaling pathways, autophagy regulation, and ligand–receptor cytokine–cytokine receptor interactions [284].

Alcohol drinking is a risk factor for developing gastrointestinal cancer by facilitating intestinal barrier dysfunction, which occurs even weeks after the cessation of alcohol intake. Ethanol exposure (acute and chronic) promotes the dysregulation of intestinal stem cells and favors long-lasting intestinal damage. Intestinal damage stimulates the division of stem cells (to regenerate the damaged epithelium); thus, the stem cells divide more frequently. These divisions can induce mutations leading to the dysfunction and transformation of intestinal stem cells [285]. Chronic alcohol intake reduces Bmi1 expression (a stem cell marker) and dysregulates β-catenin signaling (a pivotal regulator of its target Lgr5 gene and intestinal stem cell function). In the same study, organoids generated (from small intestine tissue) and in vitro exposed to alcohol (0.2%) for seven days exhibited decreased growth and diminished the expression of Bmi, p-β-catenin (ser552), and Lgr5 [285]. The above findings suggest that the dysregulation of the intestinal stem cells is a process through which alcohol (acute and chronic) promotes long-lasting intestinal injury.

Human dental pulp stem cells are multipotent mesenchymal stem cells that differentiate into osteogenic/odontogenic cells and neuronal cells through a trans-differentiation mechanism [282,286]. Ethanol promotes transcriptomic changes and affects many human dental pulp stem cells by altering the DNA methylation profiles [286,287]. During osteogenic/odontogenic differentiation, alcohol inhibited lysine-specific demethylase 6B (KDM6B) activity, and a dysregulation of the mineralization potential occurred [287]. The latter means that alcohol decreases mineral deposition, inducing an altered dental development and osteoporosis/osteopenia.

During early hepatic specification, alcohol decreased hepatic progenitor cells’ formation and proliferation of early and mature hepatocyte-like cells [288]. At a mature stage of hepatocyte-like cells, alcohol increased two liver progenitor subsets, promoted an oxidative mitochondrial injury, and induced disease phenotypes in the liver in a dose-dependent manner (hepatocellular carcinoma markers and steatosis). It seems that these phenotypes are related to an increase in oxidative stress since antioxidant treatment reversed some of them. Thus, during the fetal period, it appears that alcohol exposure impairs the generation of progenitor cells (at an early stage of hepatic specification) and decreases fetal hepatocyte proliferation. In contrast, alcohol contributes to generating disease phenotypes during the post-natal and mature phases [288]. Human-induced pluripotent stem cells differentiate into mature human cell types, including functional hepatocytes [289]. Hepatocyte-like cells obtained from human-induced pluripotent cells (iPSCs) exposed to ethanol in vitro showed decreased alpha-fetoprotein (AFP, an early hepatic marker) and apoptosis [290]. The proliferation of more mature hepatic cells diminished and lipid droplets increased [290]. Alcoholic steatohepatitis accelerates early hepatobiliary tumors (showing molecular characteristics of hepatocellular carcinoma) and increased stemness markers (CD133 and Nanog). These markers evidence epithelial–mesenchymal transition, and it appears that the alcoholic microenvironment (e.g., pro-inflammatory molecules) is responsible for the activation of progenitor cells [291].

CSCs originate new CSCs and more differentiated bulk cancer cells [292,293]. The CSC number increases after the symmetric division, leading to a high undifferentiated and aggressive state [292,294,295]. CSCs with differentiation and self-renewal capacity are involved in cancer initiation and progression, invasion and metastasis, recurrence, heterogeneity, and resistance to the conventional therapies used in clinical practice [283,296]. These cells may maintain a proliferative or quiescent state [292,294,295,297,298]. When quiescent, it is essential to note that these cells escape the immune system’s attacks and resist anticancer treatments (e.g., chemotherapy) [292,299,300]. These latter findings mean that these cells are involved in cancer recurrence and exhibit a high invasion and metastasis capacity [292,301,302]. It seems that tumors containing CSCs responsible for cancer propagation originate, after mutations, from the normal stem and progenitor cells or non-cancer stem cells suffering de-differentiation and interconversion processes [303,304]. Therefore, CSCs are a potential target to prevent cancer development. Notably, CSC are poorly differentiated or undifferentiated cells that resist standard therapies.

In contrast, differentiated tumor cells (99% of the cells located in the tumors) are more responsive to these therapies [297]. One therapeutic strategy against undifferentiated or poorly differentiated CSCs is to promote the elimination of differentiation by conventional treatments [297]. CSCs dwell in niches controlled by cells (stromal fibroblasts and immune, endothelial, and adipose cells) and components of the extracellular matrix, growth factors, chemokines, cytokines, angiogenesis, and pH [304]. Hence, some processes (e.g., wound healing, hypoxia, and inflammation) can create carcinogenesis-promoting niches [292]. Alcohol can encourage the de-differentiation of cancer cells into CSCs through microenvironment inflammatory mechanisms [292,305]. ROS generation by alcohol can alter the microenvironment of these niches and, hence, CSC functional attributes (e.g., self-renewal, survival, differentiation) [283]. Along this line, alcohol alters the extracellular matrix components by modifying the CSC microenvironment [283,291,305]. Alcohol augments the CSC population, explaining tumors promoted by ethanol [283]. In tumor-suppressor p53-deficient animals, ethanol blocks apoptosis and promotes an early appearance of dysplasia; hence, p53-dependent apoptosis counteracts the tumorigenic action exerted by ethanol [306]. In CSCs, alcohol increased the expression of the ErbB1, ErbB2, and ErbB3 epidermal growth factor receptors, and in these cells, which expressed a low level of ErbB2, the increase in the number of CSC mediated by alcohol was lower compared to CSC-overexpressing ErbB2 [283,307,308]. Thus, the proliferative activity of CSC mediated by alcohol appears to be related to ErbB2 expression.

CSCs, exposed to ROS, activate antioxidant mechanisms and show a malignant phenotype [303]. However, the generation of ROS associates with pro-survival stimulation pathways controlled by Akt/PI3K and cytokine signalings. It seems that, in CSC, the responses against the generation of ROS can stimulate survival mechanisms, maintaining their characteristics and properties [303]. In oro-esophageal squamous-cell-type carcinoma, alcohol provoked the expansion of the CSC-like population [309].

Conversely, the activation of the p38 MAPK/β-catenin pathways by alcohol promoted stem and progenitor cell expansion [310]. Alcohol prompts the migration and invasion of these cells through the ErbB2/p38γ MAPK axis in breast cancer stem cells [307] and via the Toll-like receptor 4-Nanog pathway in hepatic cancer stem cells [311]. In the liver of mice treated with alcohol, CSCs (Nanog and CD133) are upregulated, and proliferation (p53, cyclin D1, BrdU) markers increase [283]. After stimulating CSC expansion, the MAPK isoform p38γ induces the development, progression, and aggressiveness (migration and invasion) of cancer. Silencing reduces the expressions of Sox2, Oct3/4, and Nanog in CSC [312]. In an ErbB2-dependent manner, alcohol activated p38γ. It appears that the activation of the p38γ/ErbB2/discs large MAGUK scaffold protein/synapse-associated protein-97 axis mediates, in addition to migration and invasion, the increase in the number of CSC incited by alcohol [308]. RhoC (a protein belonging to the Rho family of GTPase) regulates CSC and increases when alcohol activates p38γ [313]. This activation and upregulation increases the migration and invasion of cancer cells and affects the CSC population [283,308]. In transgenic mice, long-term alcohol exposure promotes hepatic tumors. Tumor-initiating stem cell-like cells isolated from these mice show tumorigenic activity and self-renewal directed by Toll-like receptor 4 via Nanog stem cell factor’s upregulation. A transforming growth factor β pathway appears defective [279,314] and mice showing an attenuated path of this factor develop hepatic tumors.

In contrast, alcohol ingestion increased tumor incidence in a Toll-like receptor 4-dependent manner [314,315]. Thus, Toll-like receptor 4 is involved in the malignant transformation of cells. These findings show new potential therapeutic targets for the treatment of liver tumors. Toll-like receptor 4/Nanog pathway activation also promoted the degradation of p53 through the phosphorylation of NUMB (a protective protein) and its p53 dissociation by the TBC1D15 oncoprotein. This oncoprotein probably links cell self-renewal and metabolic reprogramming [316]. Because Nanog/NUMB/p53 signaling controls CSC and hepatic tumorigenesis self-renewal, a promising therapeutic method may be the targeting of protein NUMB phosphorylation [317].

Alcohol promotes endotoxemia by decreasing permeability in the gut and the inflammatory response after activating the liver Toll-like receptor 4 [317,318]. The generated endotoxins bind to the Toll-like receptor 4-CD14 complex and induce hepatoblast and hepatocyte activation and Nanog expression. These mechanisms promote the generation of chemoresistant tumor-initiating stem-like cells, developing hepatocellular carcinoma. Notably, Nanog induces fatty acid oxidation (this mechanism switches stem cell and cancer stem cells’ fates) and blocks mitochondrial oxidative phosphorylation, leading to the inhibition of oxygen consumption and ROS production [317]. These metabolic changes facilitate cell self-renewal and oncogenesis. A decrease in fatty acid oxidation and the restoration of oxidative phosphorylation normal levels decrease the CSC capacity to induce tumorigenesis and increase chemotherapy sensitivity [315,317]. Altogether, the data show that Nanog reprograms the metabolism of mitochondria and that the changes observed are responsible for the oncogenicity and chemoresistance of CSC.

Breast cancer stem cells resist radiation action, and this therapy alone fails to induce an antitumor immune response. In this regard, disulfiram/cooper rendered ionizing radiation-resistant breast CSC as sensitive as non-breast CSC to ionizing radiation-induced immunogenic cell death. Inhibition of X-box binding protein 1 spliced (XBP1s) and ROS scavengers partially reverse disulfiram/cooper-induced immunogenic cell death of breast CSC. This study showed the potential of ionizing radiation and disulfiram/cooper to promote immunogenic cell death in differentiated and differentiating breast cancer cells and radiation-resistant breast CSCs involved in cancer formation, progression, and metastasis [319].

In patients suffering from malignant hepatocellular carcinoma, ethanol augments both tumor aggressiveness and progression [320]. Alcohol consumption positively correlates with vessel invasion and TNM stage, and non-drinker patients with hepatocellular carcinoma show a slow progression rate and a better prognosis than chronic drinkers [320]. Ethanol increases the CSC population and enhances the invasion and metastasis and hepatocellular carcinoma cells’ stemness. Moreover, it promotes an epithelial-to-mesenchymal transition by activating the Wnt/β-catenin signaling pathway. The application of salinomycin (an inhibitor of the previous pathway) or β-catenin siRNA partially rescues ethanol-induced epithelial-to-mesenchymal transition. This study demonstrated that ethanol promotes metastasis and stemness of hepatocellular carcinoma cancer cells by inducing an epithelial-to-mesenchymal transition. Moreover, the Wnt/β-catenin/glycogen synthase kinase 3 β signaling pathway regulates CSCs [320]. The activation of this pathway by alcohol promotes tumorigenesis and increases the migration and invasion of cancer cells [283].

High alcohol exposure develops cancer in tissues in direct contact with alcohol (e.g., esophagus, upper larynx, pharynx, and oral cavity). Its carcinogenic action mechanism is unknown, but its metabolite, acetaldehyde, exerts a mutagenic activity [321]. A direct effect of long-term use of alcohol-containing mouthwashes suggests a possible link between oral cancer and ethanol cytotoxicity [321,322]. In keratinocytes, alcohol-induced cell death promotes the mitosis of stem cells (cell division can induce cancer-promoting errors as mutations during DNA replication) [322,323]. In a concentration-dependent manner, ethanol stimulates stem cells’ division to regenerate the damaged epithelium, increasing cancer development risk in those tissues in direct contact with alcohol [322,324]. Augmented stem cell divisions favor more DNA alterations and a higher risk of malignant transformation [323]. Notably, tissues’ self-renewal capacity relates to the risk of developing cancer. A tissue with high self-renewal capacity develops cancer more frequently than tissue with a lower capacity [325]. Additionally, alcohol intake by favoring stem cell divisions also increases the risk of cancer development in both the esophagus and pharynx and these stem cells become vulnerable to acetaldehyde and other carcinogenic tobacco components. Therefore, a non-cytotoxic concentration of ethanol may reduce the risk of esophagus, oral cavity, larynx, and pharynx cancer development and decrease tobacco smoking’s synergistic effect on these cancers [323]. Altered transcriptional regulation mechanisms are crucial in cellular transformation. Ethanol and nicotine mitigate DNA methylation and histone modifications in normal human oral keratinocytes [326]. Quiescent cells are less vulnerable to DNA-damaging agents than dividing cells. In the former, the nuclear membrane protects the DNA highly packaged into chromatin. Due to the lack of protective events in the dividing cells, DNA interacts more efficiently with DNA-damaging agents [323].

**Figure 8:**
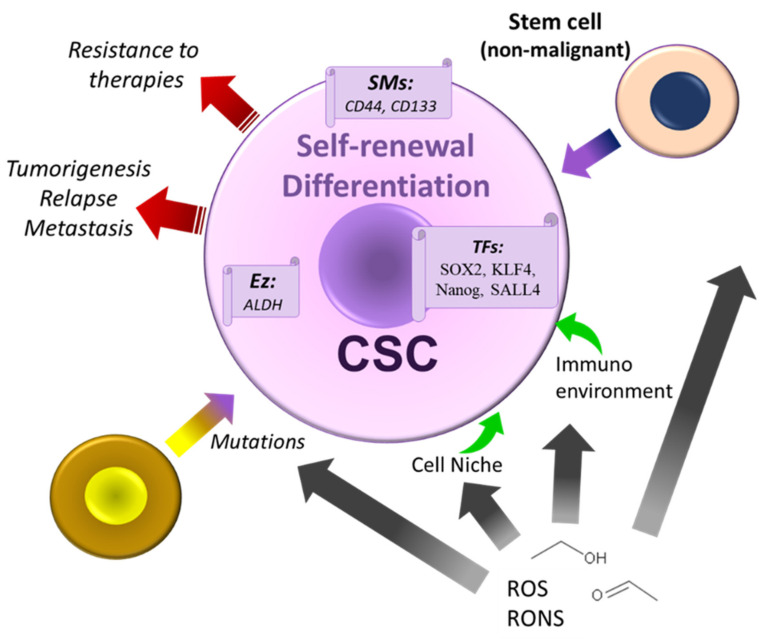
Schematic representation of a cancer stem cell (CSC) generated from non-malignant progenitor stem cells or somatic and differentiated cells by altering essential signaling pathways, epigenetic mechanisms, and mutations. Ethanol and its metabolites may interfere with critical molecular events related to signaling pathways, gene expression regulators, and environmental factors and consolidate CSC phenotypes and clonal heterogeneity within a tumor in different tissues (see text for details). Labels (Ez, SMs, and TFs) indicate markers that characterize CSCs. ALDH, aldehyde dehydrogenase; Ez, enzymes; SMs, cell surface markers; TFs, transcription factors [266,269,270,275,283,293,299,300,301,302,303,311,313,328,331,332,333,334,335].

Chronic alcohol administration promotes pancreatic cancer development by transforming human pancreatic normal ductal epithelial cells into cancer stem-like cells [327]. During the transformation process, cells showed the CSC phenotype and expressed stem cell markers (CD133, CD44, and CD24) and pluripotency-maintaining factors (KLF4, c-myc, and Sox2) [327] (Figure 8). Oct4, Sox2, and Nanog are transcription factors that negatively regulate genes favoring differentiation mechanisms [328]. SATB2 (a transcription factor that regulates the expression of pluripotency factors) can bind to the promoters of c-myc, Oct4, Bcl-2, Sox2, and *KLF4* genes. Interestingly, inhibition of the SATB2 expression blocked colony formation, CSC markers, pluripotency, and cell proliferation [327]. SATB2 overexpression was associated with cell stemness phenotype acquisition [292,329]. All data, taken together, point out that the chronic ethanol exposure of human pancreatic normal ductal epithelial cells promotes a CSC phenotype via SATB2.

Witte et al. [330] reported high global transcriptional similarities between CSCs from different cancers (prostate, uterus, lung, and brain). These cancers share upregulated gene expression related to ribosomal and mitochondrial activities. Therefore, CSCs from the tumors exhibit many similarities, which are independent of their tumor of origin. This finding is relevant and offers promising research lines for the targeting of CSCs.

CSCs (Figure 8) can arise from normal stem cells suffering an oncogenic transformation or progenitor or differentiated somatic or tumor cells suffering a de-differentiation process (de-differentiated cells acquire stemness features). Mechanisms increasing the number of CSCs also increase cancer incidence and cancer recurrence [292]. As mentioned above, the accumulation of stem cell divisions is a significant cause of cancer. It seems that cancer originates in these cells [323], which means that the mutagenic agents are not the only cause of cancer. By increasing the CSC population or inducing stemness traits in cancer cells, alcohol can prompt cancer therapy resistance, recurrence, and aggressiveness [283]. CSCs are refractory to surgical resection, radiation therapy, and chemotherapy, and a report showed that 40% of hepatocellular carcinoma derives from CSC [317]. This example shows the importance of CSCs in cancer development and recurrence. The targeting of these cells is crucial to inhibiting both processes since CSCs are chemoresistant and promote cancer recurrence. CSCs express pluripotency (stemness)-associated transcription factor networks (e.g., Notch and Wnt/β-catenin) (Figure 8). They also exhibit dysregulation in gene expression. Some signaling pathways altered, e.g., phosphatidylinositol 3-kinase/Akt, STAT3, and Sonic hedgehog, transforming growth factor β [317]. In CSCs, alcohol induces ROS (which activates Toll-like receptor 4-Nanog, Wnt/β-catenin/glycogen synthase kinase three β, and p38 MAPK signaling pathways), activates epidermal growth factor receptors, and alters the CSC microenvironment. Additional signaling pathways (e.g., transforming growth factor β and JAK-STAT) are involved in CSC proliferation. 

Given the multiple signaling pathways implicated, a combined therapeutic approach to eliminate or block CSC is imperative. The targeting of these cells within the context of alcohol consumption pertains to niches and microenvironment, surface markers, differentiation, and stemness-concerned pathways. The direct effects of ethanol and its metabolites on specific molecular targets responsible for the homeostasis of stem and progenitor cells and the molecular changes related to those effects are summarized in Table 3.

Alcohol may also act by modifying epigenetic mechanisms in stem-like and differentiated cells. In the context of alcohol exposure, the analysis of the implication of oncogenic miRNAs and other epigenetic events in cell transformation may lead to identifying possible pharmacological targets. The altered function of some miRNAs induced by alcohol intoxication and other concomitant factors relates to the formation of head and neck squamous cell carcinoma [256] or hepatic inflammation and fibrosis [336]. Additionally, alcohol may change lncRNAs’ actions and trigger cell transformation of liver stem cells, leading to hepatocellular cancer [317].

## 6. Concluding Remarks and Future Perspectives

To date, the molecular and cellular mechanisms responsible for carcinogenesis and induced by alcohol are not well-understood [149,269]. Cancers encompass heterogeneous diseases with multifactorial etiology, common characteristics, and specific features depending on the cell type and tissue affected. The relationship between alcohol and cancer occurrence rests on an intricate net of molecular events hard to isolate. To decipher whether alcohol is the principal causal effect for a given type of cancer requires further study. Moreover, alcohol shares etiological impacts with other environmental factors and individual genetic backgrounds contributing to tumor development and progression.

This review provided a detailed analysis based on in vivo and in vitro experiments of metabolic pathways that suffer modifications and detours after ethanol exposure. The reactivity of oxidation products, such as acetaldehyde and free radicals generates DNA adducts that induce genetic instability and may accentuate oncogenic machinery. Consequently, somatic and stem cells accumulate mutations eventually responsible for tumorigenesis. Additionally, nonoxidative metabolites that influence membrane dynamics may compromise membrane function. Conversely, the imbalance in microbiome communities in the oral cavity and gastrointestinal tract observed after ethanol presence reinforces direct damage through bacterial metabolites affecting the system’s normal function and generating a leaky gut that paves the way for inflammation events. Human population studies offer valuable data concerning the influence of polymorphisms on the impact of alcohol. The analysis of human tumor samples offers the possibility of ascertaining molecular changes linked to alcohol consumption.

We propose further studies on the influence of ethanol metabolism using different cell cultures and experimental animal approaches (chronic, acute, and binge exposure, both in vitro and in vivo). In addition, studies on the role of critical enzymes involved in ethanol oxidation and other metabolic routes, including antioxidant pathways or reactions within the one-carbon metabolism hub, might provide a clearer picture. Moreover, the study of signaling pathways, such as TLR4/Nanog, EGFR/ErbB2, *TP53*, *APC,* or *KRAS,* will provide insight into visualizing how and to what extent ethanol leads to cell proliferation and malignancy.

The evidence relating direct alcohol biochemical damage with the onset of cell transformation is not conclusive. However, the strong indication provided by basic and clinical research sets ethanol and its metabolic realm as crucial etiological elements. It prompts the detailed analysis of some targets with increased sensitivity to ethanol or its metabolites that may play a predominant role in the etiopathogenesis of some cancers in a specific patient. Clinical and molecular views must collaborate to define profiles of cancers for designing tailored therapeutic strategies. Table 4 presents some targets and lines of research to design new and more effective therapies to tackle different cancers.

Alcohol is an avoidable external agent. Therefore, the design of prevention programs should prioritize contributing to better health standards and reducing healthcare expenses. The latter assertion may sound naïve since alcohol drinking tendency and behavior lie in deep domains of interconnected human genetic backgrounds, human cultural traditions, and social behavior.

## Figures and Tables

**Table 1:** Representative examples of enzyme variants liaised to cancer risk participating in alcohol metabolism or other metabolic pathways related to alcohol metabolism. The four EC digits represent the nomenclature assigned by the Enzyme Commission (EC) of the International Union of Biochemistry and Molecular Biology (IUBMB) [196] (accessed on 1 April 2021). The nomenclature rs (RefSNP) refers to gene variants named after The Single Nucleotide Polymorphism Database (dbSNP) [197] (accessed on 21 March 2021). HSD17B13, hydroxysteroid 17-beta dehydrogenase; MS, methionine synthase; MTHFR, methylenetetrahydrofolate reductase; MTRR, methionine synthase reductase; PNPLA3, patatin-like phospholipase domain-containing protein 3 or adiponutrin; GSH, reduced glutathione; TM6SF2, transmembrane 6 superfamily member 2; SNV, single nucleotide variants. * indicates that the single nucleotide polymorphism (SNP) is a single variant, SNV, appearing in the population with a 1% frequency or higher.

Enzyme	SNVs *	Gene Variants and Cancer Risk References
ADH, EC 1.1.1.1	rs1229984, rs2066702, rs698, rs1693482	[25,50,175,176,177,180,189,198,199,200]
ALDH, EC 1.2.1.3	rs671	[50,175,177,180,188,191,198,200,201,202,203,204]
CYP2E11, EC 1.14.14.1	rs2031920, rs6413432	[55,176,193,205]
HSD17B13, EC 1.1.1.51	rs 4607179	[206]
SOD, EC 1.15.1.1	rs4998557, rs4880	[207,208,209]
PNPLA3, EC 2.3.1.51	rs738409	[69,206,210,211,212,213]
MTHFR, EC 1.5.1.20	rs 2184227, rs868014	[174,179,214]
MS, EC 2.1.1.13	rs1805087	[215]
MTRR, EC 1.16.1.8	rs162036, rs1532268	[213,215]
TM6SF2	rs58542926	[213]

**Table 2:** Representative targets of ethanol and its metabolites deserving further research to establish ethanol’s participation in cancer development and progression and design new pharmacological tools.

Target	Mechanism	Effect	References
ADH	Increased demand by substrate presence	[NAD^+^]/[NADH] imbalance	[48,49,50]
ALDH	Increased demand by substrate presence	[NAD^+^]/[NADH] imbalance	[60]
Catalase	Increased demand by substrate presence	ROS production	[51,201]
CYP2E	Increased demand by substrate presence	ROS production	[53]
Fatty acid ethyl ester (FAEE) synthase and carboxyl ester lipase (CEL)	Increased demand by substrate presence	Accumulation of fatty acid ethyl esters (biomarkers of alcohol consumption). It alters AMP kinase (AMPKα) activity	[41,46,89,93]
Sulfotransferase (ST)	Increased demand by substrate presence	Accumulation of ethyl sulfate (a biomarker of alcohol consumption)	[95]
UGT	Increased demand by substrate presence	Ethyl glucuronide (a biomarker of alcohol consumption) accumulates; it activates Toll-like receptor proteins	[38,94]
PLD	Increased demand by substrate presence	Phosphatidylethanol (a biomarker of alcohol consumption) accumulates; it produces changes in membranes and alters the phosphatidic acid synthesis	[33,36]
PARP (poly (ADP-ribose) polymerases]	[NAD^+^]/[NADH] imbalance	Defects in DNA repair	[106]
Sirtuins	[NAD^+^]/[NADH] imbalance	Loss of histone deacetylation	[105]
AMPK	[NAD^+^]/[NADH] imbalance	Alteration in energy equilibrium and anabolic metabolism, cell growth, proliferation, autophagy, and DNA repair	[107]
MS	Inhibition by acetaldehyde	Downregulation of the conversion of methyltetrahydrofolate to tetrahydrofolate and synthesis of methionine	[268]
NADPH oxidase and inducible nitric oxide synthase (iNOS)	Activation by acetaldehyde	ROS formation	[152,153]
Superoxide dismutase (SOD)	Downregulation/upregulation by ethanol and acetaldehyde	Imbalance of ROS	[160,161]
Lipids, proteins, and DNA	Formation of adducts with acetaldehyde, ROS, and HNE	Lipid peroxidation, dysregulation of protein function, and disruption of gene expression	[62,80,82,83,149,229,231]

**Table 3:** Summary of observations that link alcohol exposure with stem and progenitor cells changes, leading to cancer development, cancer progression, and resistance to current and conventional treatments (see text for details).

Actors	Mechanisms	Effects	References
Long-term alcohol treatment	Upregulated expression of malignant genes (e.g., *TP63, KRT15, SAMD9, STEAP4, ITGB6*)	Oncogenic transformation and appearance of breast cancer	[269]
ROS imbalance	Biomembranes, DNA, and protein damage	Affects differentiation/self-renewal of different types of stem cells	[268,271,272]
Ethanol and acetaldehyde	Alteration in epigenetic mechanisms (DNA methylation, histone modifications, and noncoding RNAs)	Dysregulation of self-renewal and differentiation pathways in embryonic and adult stem cells	[275,276,277,285,286]
Ethanol and its metabolites	Changes in stem cell niche’s microenvironment, modification of membrane fluidity, and derangement of the extracellular matrix composition and organization	Block of cell differentiation, DNA damage, and promotion of apoptosis	[268]
Acute and chronic treatments	Activation of intestinal stem cell divisions and dysregulation of β-catenin signaling	More frequent divisions cause mutations and malignant cell transformation	[284]
ROS and acetaldehyde (mitochondrial oxidative overload)	Alteration of hepatic progenitor cells formation and differentiation	Development of steatosis and hepatocarcinoma	[287,288,289]
ROS imbalance	Modification of CSC microenvironment and control of ErbB2 expression	Increased number of CSCs, augmented cell survival and potentiated malignity, and tumor propagation	[268,306,307,337]
ROS imbalance	Activation of the p38 MAPK/β-catenin pathways	Promotion of stem and progenitor cell expansion	[309]
High alcohol exposure	Direct cytotoxic effect or through acetaldehyde-induced mutations	Development of tumors of the oropharynx and gastrointestinal tract (mechanisms not well understood)	[320]
Chronic alcohol drinking	Promotes transformation (alteration of stemness markers)	Human pancreatic ductal epithelial cells transform into cancer stem-like cells	[338]

**Table 4:** Representative examples of candidate targets for developing new treatments to reverse ethanol’s damaging effects on cell development, proliferation, and metabolism homeostasis associated with cancers.

Target	Tumor (s)	Therapeutics Design	References
Acetaldehyde and ROS formation. Mouth and gut microbiomes.	The gastrointestinal tract, oropharynx, lung, liver, and other tissues.	Reduction in acetaldehyde and acetaldehyde reactivity and regulation of glutathione concentrations. Antioxidant strategies to quench free radicals and modulation of antioxidant enzymes: catalase, glutathione S-transferase, glutathione peroxidase, and glutathione reductase. Control of microbiome.	[13,339,340,341,342,343]
The hub of one-carbon metabolism.	Colorectal and other tissues.	Increased plasma concentrations of betaine and methionine.	[344]
Regulation of NAD synthesis.	Stomach, skin, and other tissues.	Diet supplement of nicotinamide riboside and nicotinic acid riboside and enzymatic regulation of nicotinamide phosphoribosyltransferase (NAMPT) and nicotinamide mononucleotide adenylyltransferase (NMNAT).	[345,346,347]
PARP/Sirtuins.	Liver, breast.	Development of specific PARP inhibitors.	[106,348,349]
NUMB protein.	Breast, liver.	Targeting phosphorylation at specific amino acid residues.	[316]
Wnt/β-catenin/glycogen synthase kinase 3-β signaling pathway.	Liver	Inhibition of β-catenin with siRNA.	[319]
EGFR and ErbB2.	Breast.	Targeting phosphorylation.	[337]
TLR4/Nanog signaling.	Liver.	Control of oxidative phosphorylation and fatty acid oxidation.	[316]
Long-noncoding RNAs.	Liver, esophagus, ovary.	Control of expression of long noncoding RNAs.	[316]
microRNAs.	Liver.	Drugs aimed at blocking microRNAs involved in liver fibrosis (for example, microRNA-378 and miR-34c).	[336,350]
Critical proteins in CSC: Notch, Wnt, Hippo, Hedgehog, PIK3/Akt/mTOR.	Several tissues.	Immuno-control and -regulation of activity through reversible and irreversible covalent changes of signaling routes and transcription factors responsible for uncontrolled stem role.	[265,268,274,283,291,298,299,300,301,302,310,312,327,329,331,332,333,334,337]
